# Oncogenetics service and the Brazilian public health system: the experience of a reference Cancer Hospital

**DOI:** 10.1590/1678-4685-GMB-2014-0364

**Published:** 2016-05-13

**Authors:** Edenir I. Palmero, Henrique C.R. Galvão, Gabriela C. Fernandes, André E. de Paula, Junea C. Oliveira, Cristiano P. Souza, Carlos E. Andrade, Luis G.C. Romagnolo, Sahlua Volc, Maximiliano C., Cristina Sabato, Rebeca Grasel, Edmundo Mauad, Rui M. Reis, Rodrigo A.D. Michelli

**Affiliations:** 1 Centro de Pesquisa em Oncologia Molecular, Hospital de Câncer de Barretos, Barretos, SP, Brazil; 2 Faculdade de Ciências de Saúde de Barretos, Dr. Paulo Prata - FACISB, Barretos, SP, Brazil; 3 Departamento de Oncogenética, Hospital de Câncer de Barretos, Barretos, SP, Brazil; 4 Life and Health Sciences Research Institute (ICVS), Health Sciences School, University of Minho, Braga, Portugal; 5 ICVS/3B's-PT Government Associate Laboratory, Braga/Guimarães, Portugal

**Keywords:** oncogenetics, hereditary cancer, public health

## Abstract

The identification of families at-risk for hereditary cancer is extremely important due to the prevention potential in those families. However, the number of Brazilian genetic services providing oncogenetic care is extremely low for the continental dimension of the country and its population. Therefore, at-risk patients do not receive appropriate assistance. This report describes the creation, structure and management of a cancer genetics service in a reference center for cancer prevention and treatment, the Barretos Cancer Hospital (BCH). The Oncogenetics Department (OD) of BCH offers, free of charge, to all patients/relatives with clinical criteria, the possibility to perform i) genetic counseling, ii) preventive examinations and iii) genetic testing with the best quality standards. The OD has a multidisciplinary team and is integrated with all specialties. The genetic counseling process consists (mostly) of two visits. In 2014, 614 individuals (371 families) were seen by the OD. To date, over 800 families were referred by the OD for genetic testing. The support provided by the Oncogenetics team is crucial to identify at-risk individuals and to develop preventive and personalized behaviors for each situation, not only to the upper-middle class population, but also to the people whose only possibility is the public health system.

## Introduction

Genetic counseling in the oncology context is a process of communication that deals with problems associated with genetic disorders, and is directed to individuals and families suspected of having a cancer predisposition syndrome (INCA, 2009). It is estimated that 5% to 10% of all tumors are caused by inherited genetic alterations that confers to the carrier a significantly higher cancer risk than the general population. Thereby, the identification of individuals at-risk for hereditary cancer is important for several reasons: *i*) affected individuals have a cumulative vital risk (CVR) much higher than the population for various types of cancer; *ii*) other relatives of an affected individual may be at-risk too; and *iii*) intensive screening measures followed by preventive intervention (chemoprevention and prophylactic surgery) are significantly effective in reducing the risk of cancer in mutation carriers (Rebbeck, 1999; Eisen *et al.*, 2000, 2005; Hartmann *et al.*, 2001; Kauff *et al.*, 2002; Rebbeck *et al.*, 2002).

In Brazil, multidisciplinary care of clinical genetics has historically received little attention by both public and private Brazilian health systems (Goss *et al.*, 2013; Horovitz, 2013). This perspective is slowly changing due to: *i)* increased scientific knowledge of the diseases, *ii)* civil society mobilization and *iii)* government actions. As examples of this improvement, we could mention the establishment of the *National Policy on Comprehensive Care for People with Rare Diseases* (BRASIL, 2014) or the more clear, although restrictive, resolutions for genetic test coverage of private health insurance, ruled by the *Supplementary Health National Agency* (BRASIL, 2013).

Notwithstanding these achievements, healthcare for late onset hereditary diseases, such as the majority of the familial cancer predisposition syndromes, are still on the periphery of discussions. The number of Brazilian genetic services providing oncogenetic care is extremely low considering the continental dimension of the country and its approximately 200 million people (Palmero *et al.*, 2007a). Additionally, those few health services are mostly located in the capitals of some Brazilian states, thus limiting or, at least, hindering access to the population living in outlying areas. Therefore, patients who might be at high risk do not receive appropriate assistance (non directive pre- and post-test genetic counseling - ASCO; Robson *et al.*, 2010). As a consequence, very little is known about the type of patient and families that seek cancer genetic counseling in Brazil or whether the existing services are in fact reaching individuals at high risk for cancer predisposition syndromes (Palmero *et al.*, 2007b). Taking all of this into consideration, the present report intends to describe the creation, structure and management of a cancer genetics service established in 2010, in a reference center for cancer prevention and treatment located in the rural area of São Paulo state.

## Subjects and Methods

### Structure of the participating Reference Cancer Center

The Barretos Cancer Hospital (BCH) is a reference cancer hospital located in the countryside of São Paulo state and has a very broad coverage, receiving patients from all Brazilian states and more than 70% of São Paulo state. The Oncogenetics Department (OD) of BCH is composed by a multidisciplinary team with extensive experience in hereditary cancer. Patients seen in the OD herein described come exclusively from other internal Departments of the BCH. After consultation, patients receive all the clinical and surgical follow up, according to the risk identified through genetic counseling and genetic testing. One of the differentials of this service is the fact that the institution offers, free of charge, to all patients who meet pre-established clinical criteria (and to all interested relatives), the possibility of pursuing genetic testing, following the highest standards of quality and using the gold standard techniques for such tests.

The study was approved by the Institutional Review Board of the BCH (approval number 745/2013) and all patients included fulfilled the written informed consent.

### Clinical criteria for genetic testing

Family selection for genetic testing is made by the clinical geneticists through pedigree analysis, using the clinical criteria defined by the OD.

Regarding Hereditary Breast and Ovarian Cancer Predisposition Syndrome the criteria are: i) three or more cases of breast and/or ovarian cancer < 50 years old; ii) four relatives diagnosed with breast cancer at any age (at least two first degree relatives); iii) two cases of breast cancer < 40 years old; iv) male breast cancer and family history of ovarian or breast cancer at a young age; v) Ashkenazi ethnicity with breast cancer < 60 years old; vi) bilateral breast cancer < 50 years or bilateral breast cancer at any age and a first or second degree relative with breast cancer < 60 years; vii) breast and ovarian cancers in the same subject; viii) mutation carrier probability > 20% according to BOADICEA (Antoniou *et al.*, 2004), Myriad (Frank *et al.*, 2002), Penn II (www.afcri.upenn.edu/itacc/penn2) or Manchester (Evans *et al.*, 2004) models; ix) triple negative breast cancer diagnosed before the age of 50; x) medullary carcinoma diagnosed before 50 years old; xi) two or more relatives with pancreatic and/or prostate cancer (Gleason ≤ seven) at any age.

For Lynch syndrome, Bethesda (Boland *et al.*, 1998) and Amsterdam (Vasen *et al.*, 1999) criteria are used. For Li-Fraumeni and Li-Fraumeni *like* syndromes, the original criteria described by Li and Fraumeni (Li *et al.*, 1988), Birch (Birch *et al.*, 1994) and revised Chompret (Tinat *et al.*, 2009) are used. For Hereditary Breast and Colorectal Cancer and Cowden's syndrome, the criteria described by Meijers-Heijboer *et al.* (2003) and Nelen *et al.* (1996), respectively, are used. Regarding medullar thyroid cancer, all patients, irrespective of age and sex are referred for testing. All patients with a clinical diagnosis of Familial Adenomatous Polyposis or Attenuated Familial Adenomatous Polyposis are also referred for genetic testing. For diffuse gastric cancer, criteria proposed by the International Gastric Cancer Linkage Consortium (IGCLC) are followed.

## Results

### The Oncogenetics Department of the Barretos Cancer Hospital

Barretos Cancer Hospital, located in the city of Barretos, countryside of São Paulo state was founded in 1962 to attend oncological patients from the interior and rural areas of São Paulo. Currently, it is a tertiary referral center for the treatment of cancer in Brazil with about 11,000 new cancer cases per year, 100% covered via the Brazilian Public Health System (SUS) (Carneseca *et al.*, 2013). Patients come from more than 450 cities of São Paulo state (72.5% of the state) and 1,300 cities from other Brazilian states (21.7% of Brazilian cities). Since 1994, the BCH was concerned not only with curative measures but also began acting in cancer prevention, through screening of asymptomatic individuals (for breast, uterus, colon, skin and prostate cancer). The Prevention Department, which initially served only Barretos has expanded its coverage to other regions of São Paulo state, as well as to other Brazilian states from the North, Northeast and Midwest of Brazil, including the states Mato Grosso, Mato Grosso do Sul, Minas Gerais, Goiás, Bahia and Rondônia (Mauad *et al.*, 2002, 2010, 2011a, 2011b; Faria *et al.*, 2010). Today the Department of Prevention operates through two main fronts: i) nine mobile units (trucks) travelling throughout the State of São Paulo and to several states in the Midwest, North and Northeast of Brazil, conducting cancer screening for breast, cervical, prostate and skin and, ii) six fixed units, located in Barretos (São Paulo) (*Ivete Sangalo Prevention Institute*); Juazeiro (Bahia), Porto Velho (Rondônia), Fernandópolis (São Paulo), Campo Grande (Mato Grosso do Sul) and Nova Andradina (Mato Grosso do Sul). It is worthy of note that, in all the cities where the mobile unit circulates, its coverage is more than 60% of the local population, thus covering a representative sample of the population in the screened areas.

The awareness of the potential role of oncogenetics on cancer prevention led in 2010 to the creation of the OD at the BCH Prevention Unit. Before its creation, families at risk for hereditary cancer had a special weekly medical consultation that was basically focused on high-risk breast and ovarian cancer patients, which was further expanded to other specialties. Thus, in 2010, with the inauguration of the new Cancer Prevention Institute and its Molecular Oncology Research Center, the Oncogenetics team had the necessary structure to provide cancer risk assessment and genetic counseling, carry out preventive examinations for patients and relatives at risk for hereditary cancer and to perform genetic testing for patients and families with the best quality standards. At the present moment, the OD works together with several specialties and health care professionals as a multidisciplinary workstation.

Nowadays, the OD of the BCH is part of the *Brazilian Familial Cancer Network* and its main purpose is focused in three areas: to diagnose hereditary cancer predisposition syndromes; to perform cancer risk assessment and genetic counseling (GC); and to guide patients and families on decisions of early diagnosis and prevention.

### Structure and Work Flow

The OD offers a multidisciplinary approach (team composed by clinical and molecular geneticists, oncologists, pathologists, nurse and a psychologist) and is integrated with all specialties. The consultations are directed to patients/families referred by physicians from other specialties of BCH (it is not open to external patients).

The genetic counseling process at BCH consists (mostly) of two visits. In the first appointment, the oncogenetics nurse collects information regarding habits and health of the patients, as well as information about their families (first, second and third degree) to drawn the pedigree. Then, the geneticist confirms family data and estimates the cumulative risk of cancer (using Ibis, Gail and Claus models), as well as the previous probability to be carrier of a deleterious mutation (for families suspected of Hereditary Breast and Ovarian Cancer predisposition syndromes for example, Boadicea, Manchester, Myriad prevalence tables and Penn II models are used). In addition, information regarding cancer risk (cumulative risk, types of cancer related to the syndrome under suspect), updated information in the medical literature on cancer genetics, prevention and cancer risk reduction options are explained to the patient.

At this time, genetic testing is offered to patients with pre-established clinical criteria and that demonstrate willingness to be tested. The blood drawn (for genetic testing) can be performed on that date or in the next appointment, always under informed consent explanation. As stated above, the workflow is dynamic and can vary according to the needs of each patient.

In the second visit (or third visit, depending on the situation), the geneticist will address the genetic testing results (post-test genetic counseling), as well as the orientations for monitoring and prevention (prophylactic surgeries and follow-up tests) for patients and their families. Later follow-up of each individual may vary monthly, semi-annual and annual or eventually no more visits will be scheduled. It is important to highlight that psychological support is intended to recognize and organize possible uncertainties arising from the situation of conflict installed and assess how the patient is experiencing this situation. The Department receives professional assistance from the Department of Psychology.

**Figure 1:**
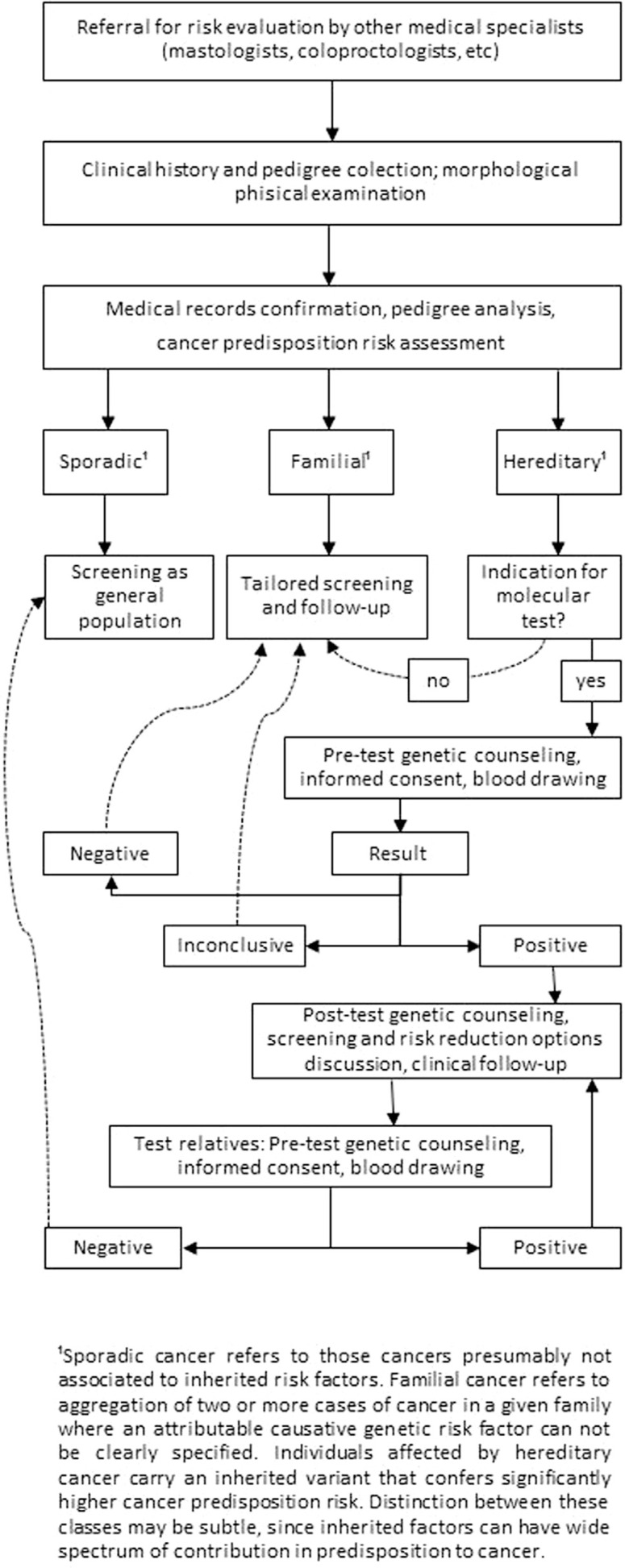
Stages of the Genetic Counseling process at Barretos Cancer Hospital (adapted from Palmero *et al.*, 2007a).

The flowchart (Figure 1) summarizes the stages of the genetic counseling process at BCH.

### Main referred syndromes

#### Hereditary Breast and Ovarian Cancer Predisposition syndrome (HBOC)

Familial breast cancer typically occurs in people with an unusual high number of family members affected by breast, ovarian or a related cancer. In these cases a differentiated management must be adopted. Therefore, it is extremely important to recognize these at-risk families and refer them to genetic counseling.

The great majority of breast cancer patients seen at the OD are referred by the Mastology Department due to a personal or family history suggestive of HBOC. In addition, patients with triple-negative tumors are also referred. Most of those patients fulfill the clinical criteria defined by the National Comprehensive Cancer Network (NCCN).

Regarding ovarian cancer, the recognition of ovarian cancer survivors at risk for HBOC syndrome is extremely important to prevent this lethal neoplasia in relatives with *BRCA* mutations. Taking this into consideration, all patients referred to the Gynecologic Oncology Department have their data collected in the first appointment during which a six-item questionnaire is employed: i) histology of dilatation and curettage, ii) age, iii) presence of multiple primary tumors, iv) family history of HBOC-related tumors, v) relatives with history of cancer before 50 years of age and vi) family with confirmed HBOC syndrome.

After answering the questionnaire and completion of the primary cancer treatment, patients with high grade serous, endometrioid or undifferentiated carcinoma histotypes, independent of their age at diagnosis are contacted to schedule an appointment at the OD. If they agree, the following steps are to participate in genetic counseling sessions and to perform *BRCA1/2* genetic testing.

#### Lynch syndrome and Familial Adenomatous Poliposis (FAP)

Concerning Lynch and FAP syndromes, the OD receives patients referred from the High Digestive Tract Cancer Department and also from the Low Digestive Tract Cancer Department, which are suspected of being carriers of cancer predisposing syndromes. Most of those patients fulfill clinical criteria well established in the medical literature for genetic counseling (Amsterdam and Bethesda criteria for Lynch syndrome) or have a hereditary cancer predisposition syndrome clinically diagnosed (which occurs in FAP syndrome due to the presence of colonic polyps).

In addition, around 5% of uterine (or endometrial) cancer is due to a hereditary cause, and most of these cases are related to Lynch syndrome. Similarly to what is performed for ovarian cancer identification, all patients referred due to uterine cancer have their data collected in the first appointment at the Gynecologic Oncology Department. A six-item questionnaire is employed (histology of dilatation and curettage, age, other primary cancer, family history of Lynch-related tumors, relatives with history of cancer before 50 years of age and family with confirmed Lynch syndrome). Patients with endometrioid, undifferentiated or clear cell histotypes younger than 60 years or with at least one relative with Lynch syndrome-related tumors are contacted after completing their primary treatment to invite to schedule an appointment at the OD.

Upon acceptance, a session of genetic counseling is held and patients younger than 60 years of age, as well as those who had at least one relative with Lynch syndrome-related cancer are invited to perform genetic testing.

In a previous publication on our first 50 patients referred due to uterine cancer, 35 had endometrioid histology and two patients with undifferentiated tumors were identified using the six-item questionnaire (Andrade *et al.*, 2013). Two (5.4%) screened patients had another primary cancer (one colorectal and one pancreatic cancer). Seventeen (45.9%) patients had first-degree relatives with Lynch-related tumors. None had a relative with confirmed Lynch syndrome. The median age of the screened patients was 60 years (31-82 years), with 18 patients younger than 60 years. Thirty one were referred for genetic counseling, but three died before the consultation, four did not accept the invitation, and three did not attend the appointment (Andrade *et al.*, 2013).

#### Hereditary Diffuse Gastric Cancer Syndrome (HDGC)

Regarding the Hereditary Diffuse Gastric Cancer syndrome, the OD receives patients referred from the High Digestive Tract Cancer Department, which have diffuse gastric cancer and/or are suspected of being carriers of cancer predisposing syndromes. Most of those patients fulfill clinical criteria well established in the medical literature for genetic counseling, asproposed by the International Gastric Cancer Linkage Consortium (IGCLC) consensus for Hereditary Diffuse Gastric Cancer.

#### Li-Fraumeni (LFS) and Li-Fraumeni like (LFL) syndromes

LFS is one of the most important cancer predisposition syndromes in Brazil, due to the Brazilian founder mutation p. Arg337His in *TP53* gene (Achatz *et al.*, 2007; Palmero *et al.*, 2008; Garritano *et al.*, 2010). The protocol used includes a regular screening of the most important sites of tumors according to age, like breast cancer in adulthood and adrenocortical tumor and choroid plexus carcinoma in childhood (Villani *et al.*, 2011).

Most patients referred to the OD, due to a suspicion of LFS/LFL, come from the Mastology Department and have a very early diagnosis of breast cancer (before 35 years old). Besides, pediatric patients are also seen by a clinical geneticist at the Pediatrics Hospital, which is part of the BCH. In this way, all children with a suspicion of a cancer predisposition syndrome (as the case of adrenocortical tumors in LFS/LFL families) have a cancer risk assessment consultation with the medical geneticist and, if criteria are met, are referred for genetic testing. In addition, the OD of BCH counts with a specialized clinic for sarcoma and central nervous systems tumor's patients, where children, adolescents and adults with a personal or family history suggestive of LFS/LFL, Von Hippel Lindau syndrome (VHL) and Neurofibromatosis (NF) are evaluated.

Families with VHL syndrome are evaluated in a multidisciplinary context with screening for the malignant conditions, as hemangioblastoma and renal cancer, as well as for non-malignant situations.

Neurofibromatosis is another condition that predisposes the individual to a sort of different benign and malignant tumors. The oncogenetic intervention in these families consists in evaluating the kindred's risk, in order to perform an early diagnose, and manage the oncologic situations that could come up.

Regarding Hereditary Breast and Colorectal Cancer (HBCC), most of the families come from the Mastology and Low Digestive Tract Departments.

Until now, 18 families with thyroid carcinoma were referred by the Head and Neck Department of BCH due to a personal and/or familial history of medullary thyroid carcinoma and, after genetic counseling, were sent to genetic testing for *RET* oncogene.

In addition, patients other than those with clear syndromic criteria, but with an undefined familial aggregation of cancer are also referred to the OD, which despite not having specific diagnosis, are kept in strict screening.

BCH is also a member of the Brazilian Cooperative on Pediatric Mielodysplastic syndromes (MDS), a multiprofessional working group whose objectives are: to provide educational support, to establish epidemiological data and to offer support and orientation for diagnosis and treatment on MDS (Lopes *et al.*, 2002). Since around 30% of MDS patients carry a predisposing condition, including congenital dysmorphic syndromes, a medical genetics specialist is required for the appropriate clinical evaluation. Among these syndromes we can mention: Neurofibromatosis Type 1, Noonan syndrome, Fanconi anemia and trisomy mosaicism. To these patients we offer genetic counseling and clinical follow-up beyond that requested for the MDS treatment.

### Molecular diagnosis of hereditary cancers

In August 2010, through an Institutional effort to establish and perform on a routine basis the genetic testing for hereditary tumors, the Center of Molecular Diagnosis (CMD) of HCB was created. All methodologies currently performed at CMD were previously internally and externally validated, through international certification (by distinct international accreditation agencies, such as the European Molecular Genetics Quality Network (EMQN), and the United Kingdom National External Quality Assessment Servic*e* (UKNEQAS). To date, over 800 families with clinical criteria for different hereditary cancer predisposition syndromes were referred by the OD for genetic testing.

Families with early diagnosed breast cancer cases (before 35 years age) and *BRCA1/BRCA2* negative or inconclusive results are tested for *TP53* mutations. Furthermore, the CMD provides genetic test for *APC* and *MUTYH* genes (FAP/AFAP), *TP53* (LLS/LFL), *MLH1, MSH2, MSH6* and *PMS2* (Lynch syndrome), *CDH1* (Diffuse Gastric Cancer), *RET* (for medullary thyroid carcinoma), *VHL* (for Von Hippel-Lindau syndrome) and *PTEN* (for Cowden syndrome).

The strategy for *APC/MUTYH/TP53/PTEN/CDH1/VHL* genetic tests consists of bi-directional capillary sequencing of all coding exons followed by Multiplex Ligation Probed-dependent Analysis (MLPA) analysis. For *RET* oncogene, bi-directional sequencing of all coding exons and intronic flanking regions is performed.

For *BRCA1/BRCA2*, since 2013, thanks to a partnership with the Institute of Molecular Pathology and Immunology at the University of Porto (IPATIMUP), the strategy of genetic testing shifted from conventional (Sanger) sequencing to next generation sequencing (using the Ion Torrent platform, Applied Biossystems) (Costa *et al.*, 2013), which enables us to analyze more patients at a lower cost and in a shorter period of time. So, since 2013, *BRCA1/BRCA2* tests are performed by next generation sequencing (Ion Torrent), followed by conventional capillary sequencing for confirmation of all identified variants. In addition, MLPA is used to investigate the presence of large rearrangements in both genes.

The genetic testing for Lynch syndrome initiates with the immunohistochemistry (IHC) screening for expression of *MLH1/MSH2/MSH6* and *PMS2* with concomitant microsatellite instability (MSI) analysis, using a panel of penta-quasi-monomorphic markers previously optimized by our group for the Brazilian population (Campanella *et al.*, 2014). The flowchart below (Figure 3) illustrates in detail how genetic testing is conducted in our laboratory.

Nowadays, the main challenge that we are facing is to create gene panels, including genes other than those conventionally tested, to identify the genetic cause of the family history of cancer in those high-risk families tested negative for single gene tests. In addition, the CMD relies completely on the Institution's financial support for genetic testing consumables, staff and purchase and maintenance of equipments.

### Current situation

**Figure 2:**
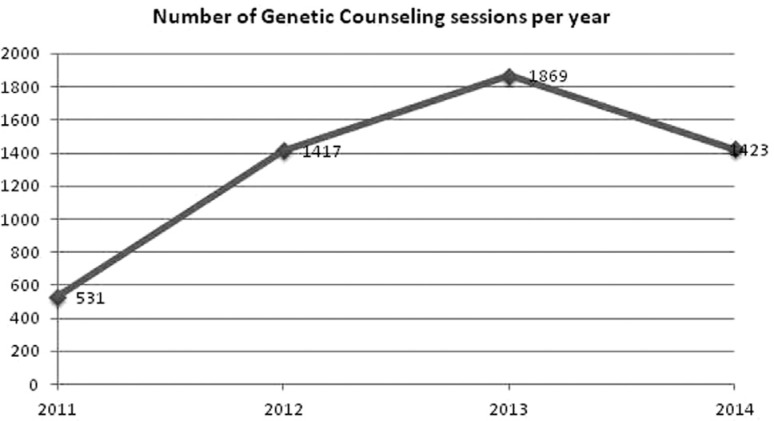
Number of Genetic Counseling sessions of the Oncogenetics Department of BCH (2014 refers to the period between January and June).

Below is described the number of consultations that have been done since 2011 (Figure 2). In 2014, between January and October, 371 patients and 243 relatives were seen by the clinical team of the OD. Seven hundred preventive exams were requested (in 2014) and were performed at BCH, including colonoscopy, mammography, breast, thyroid, abdominal and transvaginal ultrasound, endoscopy, tomography, magnetic resonance, among others.

**Figure 3:**
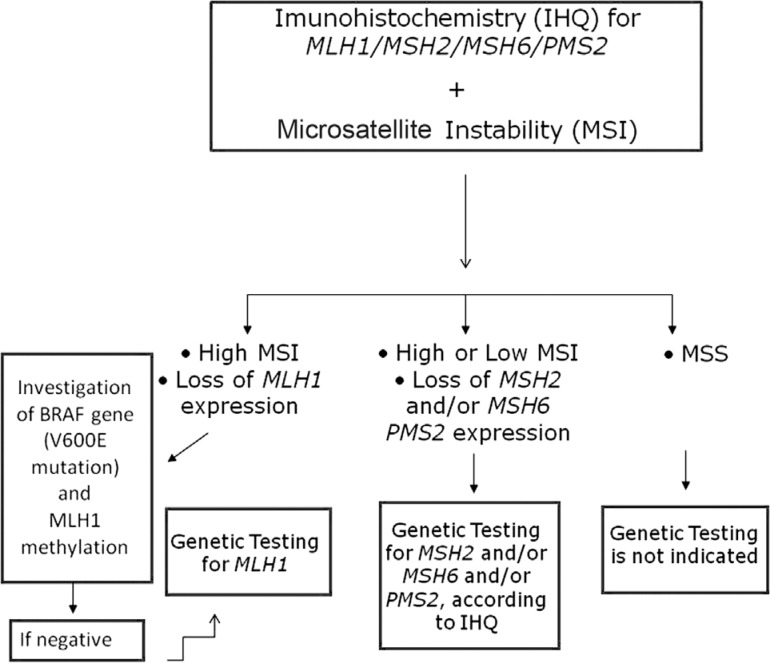
Flowchart illustrating the strategy utilized for genetic testing of patients at risk for Lynch Syndrome (flowchart adapted from Hampel *et al.*, 2008).

Regarding genetic tests, Table 1 summarizes the total number of diagnostic and predictive tests performed per year. From 2010 to 2013, 200 families were referred to *BRCA1* and *BRCA2* genetic testing. Fifty-three of these families (26.5%) were identified as carriers of germline deleterious mutations in these genes. From those 200 families, 25 were tested due to a personal and/or family history of ovarian cancer. From those, eight (32%) had mutations in *BRCA1* and one woman (4%) had a deleterious *BRCA2* mutation. In addition, we identified 44 families with FAP (deleterious mutation on the *APC* gene), 33 families with Lynch syndrome (10.8% of them with endometrioid uterine cancer), and six families with polyposis associated with the *MUTYH* gene. One family at-risk for HDGC with a variant probably deleterious was identified and is currently under follow up at the OD.

**Table 1:** Genetic tests performed at Barretos Cancer Hospital since 2011.

	BRCA1/BRCA2		IHQ^c^/MSI^d^/BRAF/MLH1/MSH2/MSH6/PMS2		APC/MUTYH		TP53		CDH1		RET		PTEN		VHL	
	IC^a^	REL^b^	IC^a^	REL	IC^a^	REL^b^	IC^a^	REL^b^	IC^a^	REL^b^	IC^a^	REL^b^	IC^a^	REL^b^	IC^a^	REL^b^
2011	64	2	61	0	21	14	5	0	4	0	0	0	0	0	1	0
2012	75	56	55	36	14	74	23	24	9	0	8	2	0	0	1	0
2013	65	129	81	69	41	90	44	38	8	4	6	0	2	0	9	10
2014	106	60	49	48	25	22	91	27	8	1	4	3	15	0	1	0

a IC: index case,

b REL: relatives,

c IHQ: imunohistochemistry,

d MSI: microsatellite instability.

Besides HBOC and Lynch, LFS/LFL is one of the main syndromes referred for genetic testing. So far, we have 30 families with deleterious germline *TP53* mutations identified, either fulfilling criteria for LFS/LFL or with a history of early breast cancer. Most of these mutations are located i the oligomerization domain of the p53 protein (exon 10, codon 337).

With respect to less common syndromes, 12 families were referred to *VHL* genetic testing. Germline deleterious mutations were identified in five (42%) of them. From the identified alterations, 80% were large rearrangements involving one or more *VHL* exons. For *RET* testing, 18 families were referred for genetic evaluation, identifying two of them as carriers of deleterious mutations. More than 15 families were referred for *PTEN* genetic testing, but none of them had deleterious mutation.

## Discussion

Family history of cancer in first-degree relatives and the presence of specific risk factors (such as bilateral cancer, individuals with multiple primary tumors, cancers at a young age, presence of several generations affected by cancer, occurrence of rare histology tumors) are important indicators of risk for hereditary cancer (Easton, 2002; Page, 2003). Recently, Carraro *et al.* (2013) evaluated 54 women with young breast cancer (younger than 35 years) and identified germline deleterious mutations in 22% of them. Considering only those patients with a positive family history of cancer, the mutated percentage increased to 43.7% (Carraro *et al.*, 2013). It is worth noting that cancer has a very negative stigma in the general population and, therefore, one should not underestimate any efforts that can generate appropriate conditions to overcome the sufferings resulting from this disease. An immediate and positive impact of the implementation of an OD is the benefit to the high risk of hereditary cancer families due to the potential for primary and secondary prevention in the families of these patients, which is possible through family genetic counseling and genetic testing.

The high potential of cancer prevention, linked to the identification of at-risk patients/families, is the reason why the OD of the BCH is hierarchically and physically located within the Prevention Unit. In addition, the institutional decision of paying for all genetic tests of high-risk patients, selected under stringent criteria, is also driven by the cancer prevention potential.

As proposed by the ASCO Policy Statement Update (Robson *et al.*, 2010), the OD of BCH is composed by a multiprofessional team which deals with clinical, psychological and social issues related to familial cancer predisposition. Because the OD team is in strict contact with other BCH Departments (Women's Health Department, High and Low Digestive Tract Cancer Departments, Head and Neck Department, Pathology Department for example), familial care begins even before patients are referred to us: interdisciplinary sessions, case presentations and faculty staff training are frequent. In fact, the Oncogenetics Department's molecular geneticists work together with pathologists. On the other hand, risk reduction procedures (such as mastectomy, colectomy or thyroidectomy) are performed by BCH experts who already were in touch with the patient and/or their family. Worth mentioning is also trained nursing, psychological support and social assistance, which are key components for high-risk familial care.

Performing the predictive genetic testing offers to the asymptomatic at-risk individuals the possibility to be included in programs for cancer prevention and early detection. Similarly, the genetic predictive testing allows the individual reassurance and eliminates waste/complications with unnecessary preventive interventions for those whose genetic test has a negative result. Despite the above mentioned, in Brazil, genetic testing for hereditary cancer predisposition syndromes is not covered by the public health system (SUS) or by most private health insurance plans, and the cost of commercially available genetic testing of private laboratories prevents its execution for the vast majority of the population. Outside the private context, genetic testing is offered within research protocols, limited to the objectives and duration of each particular research project.

Due to the high cost of genetic testing, the BCH uses stringent criteria for genetic testing indication, which can bring, as a side effect, the exclusion of at-risk families, which is a serious issue, since patients with mutations identified in the *BRCA1/BRCA2* genes, regardless of belonging to a small family, or being the only person affected by cancer in the family, have exactly the same risks for cancer development as patients from "high-risk families". Recent studies indicate that around 50% of families with mutations identified in *BRCA1/BRCA2* do not have close relatives with breast cancer or ovarian cancer (due mainly to small family size or limited family structure) and, as a consequence, do not fulfill clinical criteria for hereditary breast cancer predisposition syndrome or for genetic testing (Weitzel *et al.*, 2007).

In addition, the identification of patients with mutations in genes related to hereditary predisposition cancer syndromes is crucial for targeting specific behaviors of cancer screening, such as early diagnosis and treatment. In the case of breast cancer, for example, prospective and retrospective studies have shown that prophylactic bilateral mastectomy is the intervention with higher breast cancer risk reduction in women with a mutation in the *BRCA1/BRCA2* genes (reduction of up to 90% of the risk) (Hartmann *et al.*, 2004) Prophylactic bilateral salpingo-oophorectomy in patients with mutations promotes a 90% reduction in the risk of ovarian cancer and a 50% risk reduction for breast cancer (Roukos, 2007). In addition, regarding the newly developed target-specific therapies, we highlight the utilization of poly-adenosine diphosphate ribose polymerase-1 (PARP) inhibitors, which are specific for patients with mutations in the *BRCA* genes. Individuals at-risk for hereditary colorectal cancer may have a dramatically reduced cancer risk through an intensive screening and by performing surgery in the early stages of the disease, or even prophylactic colectomy.

In the case of medullary thyroid carcinoma it is known that conventional chemotherapeutic treatments have limited efficacy. Complete response to treatment is extremely rare and partial response is seen in only a third of the cases (Sippel *et al.*, 2008). Prophylactic thyroidectomy for at-risk individuals is the only method able to cure the patient, and the healing potential is much higher if the surgery is performed before the onset of the disease. The result of genetic testing for these patients (presence of germline mutations in the *RET* oncogene) allows the establishment of genotype-phenotype correlations and determining the best age for prophylactic thyroidectomy (Machens *et al.*, 2005).

All patients and relatives with a pathogenic germline mutation identified are under strict management at BCH. These are 53 families carrying *BRCA1*/*BRCA2* germline mutation, 33 with a mutation in Lynch-related genes, 30 *TP53* mutated, 44 with germline mutations in the *APC* gene, six with *MUTYH* associated polyposis, five with *VHL* mutations, two with MEN2A and one with HDGC. In addition, all families with negative genetic test results but with a significative family history remain under strict OD follow up. In this sense, it is important to reinforce that the support provided by the Oncogenetics multidisciplinary team is crucial, not only in identifying those at-risk individuals, but also in developing appropriate actions for each situation, thus allowing the implementation of preventive and personalized medicine, not only to the population of the upper-middle class, but also to people whose only possibility is the public health system.

To conclude, it is worthy to emphasize that a very important contribution of this work is that the results reported here can be extrapolated to similar scenarios, in national and international contexts, since services like this are scarce in Brazil and, certainly also in other countries with similar realities.
